# Impact of Genetic Background on Neonatal Lethality of *Gga2* Gene-Trap Mice

**DOI:** 10.1534/g3.114.010355

**Published:** 2014-03-17

**Authors:** Balraj Doray, Jennifer Govero, Stuart Kornfeld

**Affiliations:** Department of Internal Medicine, Washington University School of Medicine, St. Louis, Missouri 63110

**Keywords:** GGA2, gene-trap, hypomorphic allele, neonatal lethality, genetic background

## Abstract

The functional redundancy of the three mammalian Golgi-localized, γ-ear–containing, ADP-ribosylation factor-binding proteins (GGAs) was addressed in a previous study. Using insertional mutagenesis, we found that *Gga1* or *Gga3* homozygous knockout mice were for the most part normal, whereas mice homozygous for two different *Gga2* gene-trap alleles exhibited either embryonic or neonatal lethality in the C57BL/6 background, depending on the source of the vector utilized (Byg *vs.* Tigm, respectively). We now show that the Byg strain harbors a disrupted *Gga2* allele that is hypomorphic, indicating that the Byg lethality is attributable to a mechanism independent of GGA2. This is in contrast to the Tigm *Gga2* allele, which is a true knockout and establishes a role for GGA2 during the neonatal period. Placement of the Tigm *Gga2* allele into the C57BL6/Ola129Sv mixed background results in a lower incidence of neonatal lethality, showing the importance of genetic background in determining the requirement for GGA2 during this period. The *Gga2^−/−^* mice that survive have reduced body weight at birth and this runted phenotype is maintained through adulthood.

The Golgi-localized, γ-ear–containing, Arf-binding proteins (GGAs) are a family of monomeric clathrin adaptor proteins that function in receptor trafficking between the *trans*-Golgi network (TGN) and endosomes (Bonifacino 2004; Braulke and Bonifacino 2009). There are three homologous GGAs in mammalian species termed GGA1, GGA2, and GGA3. These proteins have identical domain organizations with an N-terminal VHS (VPS-27, Hrs, and STAM) domain followed by a GAT (GGA and Tom1) domain, a connecting hinge segment, and a C-terminal GAE domain that is homologous to the ear domain of γ-adaptin (Braulke and Bonifacino 2009). The VHS and GAT domains of the three human GGAs are conserved with 60–75% identity (Boman *et al.* 2000), but the similarity between each GGA becomes much less pronounced across the entire molecule within a given species. However, the individual GGAs display approximately 90% identity between the human and mouse counterparts (Govero *et al.* 2012).

We and others have noted a number of similarities between GGA1 and GGA3 that are not shared by GGA2. For instance, human GGA1 and GGA3, but not GGA2, are phosphorylated *in vivo* and subject to autoinhibition mediated by binding of internal acidic cluster-dileucine motifs present in the hinge to the ligand binding site on the VHS domain (Doray *et al.* 2002; McKay and Kahn 2004). In addition, the GAT domains of human GGA1 and GGA3, but not GGA2, bind ubiquitin and ubiquitinated proteins (Shiba *et al.* 2004; Yogosawa *et al.* 2006), and GGA1 and GGA3, but not GGA2, are depleted in the brains of patients with Alzheimer disease (Walker *et al.* 2012). Also, GGA2 has a shorter half-life than GGA1 and GGA3 (Hirst *et al.* 2007). Finally, only GGA2 is detectable in isolated HeLa cell clathrin-coated vesicles (CCVs) by either Western blotting or mass spectrometry and is dependent on AP-1 for incorporation into CCVs (Hirst *et al.* 2009; Hirst *et al.* 2012). Taken together, these findings suggest that GGA1 and GGA3 may perform more overlapping functions *in vivo* than does GGA2, and/or that GGA2 may perform some unique role not mediated by GGA1 and GGA3.

Consistent with this idea, we previously showed that loss of either GGA1 or GGA3 is well-tolerated in mice, indicating that the remaining members are able to compensate for the loss (Govero *et al.* 2012). However, gene-trap disruption of the *Gga2* gene resulted in either embryonic lethality with the BayGenomics (Byg) gene-trap or neonatal lethality with the Texas Institute for Genomic Medicine (Tigm) gene-trap (Govero *et al.* 2012). We initially considered whether this difference in phenotype was attributable to differences in genetic backgrounds, because the Byg strain was generated in the C57BL6/Ola129Sv mixed genetic background, while the Tigm strain was in the C57BL/6NJ background. However, backcrossing of the Byg strain into the C57BL/6J background still resulted in embryonic lethality, leaving the basis for this difference in outcome unclear at the time and the question of a role for GGA2 in embryonic development unresolved (Govero *et al.* 2012).

Subsequently, on review of the Western blot data of brain tissue from wild-type (wt)and heterozygous (het) progeny of the *Gga2^+/−^* crosses corresponding to the Byg strain, we observed that the het mice frequently expressed GGA2 protein at a level similar to wt, indicating that the Byg *Gga2* gene-trap allele is likely to be a hypomorphic allele. To verify this, we have generated compound het mice by crossing Byg and Tigm *Gga2^+/−^* mice in the C57BL/6J and C57BL/6NJ backgrounds, respectively. This has allowed us to unequivocally show that the embryonic lethality associated with the Byg *Gga2* gene-trap allele is not attributable loss of GGA2 because this allele is clearly hypomorphic. In addition, we introduced the Tigm *Gga2* gene-trap allele into the C57BL6/Ola129Sv mixed genetic background and demonstrated that the severity of the neonatal lethality associated with this allele is strongly influenced by the genetic background of the mice in which the allele occurs. These studies show that the *Gga2^−/−^* mice have a runted phenotype.

## Materials and Methods

All protocols involving the use of animals were in compliance with the National Institutes of Health’s *Guide for the Care and Use of Laboratory Animals* and approved by the Animal Studies Committee in the Division of Comparative Medicine at Washington University School of Medicine in St. Louis (Protocol #20130010). Mice were housed in a barrier facility maintained under standards meeting federal, state, and local guidelines and under the supervision of licensed veterinarians.

### Generation of *Gga* knockout mice

The Byg (cell-line ID SYA176) and Tigm (cell-line ID IST10483E10) *Gga2^+/−^* gene-trap mice in the C57BL/6J and C57BL/6NJ backgrounds, respectively, have been described (Govero *et al.* 2012). Primer sequences used for genotyping of mice and distinguishing between the two mutant *Gga2* alleles, PCR conditions, and the sizes of all PCR products have been presented (Govero *et al.* 2012). The screen used in this study to identify the compound hets is illustrated in Supporting Information, Figure S1.

### Harvesting mouse tissues and Western blot analyses

Adult mice were killed by CO_2_ inhalation whereas newborns were killed by decapitation. For serum preparation, blood was collected in a microfuge tube immediately after decapitation of newborns, allowed to sit at room temperature for 30 min, and centrifuged at 2000×*g* for 10 min to separate the clotted components. Brain tissue was removed and cellular extracts were prepared exactly as described (Govero *et al.* 2012). All protein samples were diluted to 5 mg/ml before being boiled in SDS sample buffer for gel loading. Proteins were resolved by SDS-PAGE, transferred to either nitrocellulose or PVDF membrane, and probed with the following antibodies: anti-GGA2 (GGA2 E-3; Santa Cruz); anti-GAPDH (Sigma); anti-CI-MPR (Zhu *et al.* 2001); anti-SorLA (BD Transduction Laboratories); and anti-Sortilin (Sigma),.

### Serum IGF-II measurement by ELISA

Serum IGF-II levels of newborn mice were measured using a modification of the ELISA procedure that has been described by Mason *et al.* (2011). All reactions were performed in 96-well tissue culture plates. Briefly, purified monoclonal rat anti-mouse IGF-II antibody (R&D) was coated on the plate [2.5 μg in a final volume of 200 μl phosphate-buffered saline (PBS) per well] overnight on a shaker at 4°. The next day, the wells were washed three times with wash buffer (PBS with 0.05% Tween-20), followed by three washes with SuperBlock T20 blocking buffer (Thermo Scientific).

For serum preparation, 100 μl acid/ethanol reagent (12.5% 2N HCl, 87.5% ethanol) was added to 15 μl serum sample and incubated at room temperature for 30 min, followed by centrifugation at 10,000×*g* for 10 min. All the supernatant was removed and neutralized with 120 μl neutralization buffer (4× PBS containing 0.25% BSA and 500 ng/ml IGF-I). Standards were prepared by diluting recombinant mouse IGF-II (R&D) in dilution buffer (PBS with 0.1% Tween-20 and 0.25% BSA). Standards ranged in concentration from 2000 pg/ml to 125 pg/ml. A blank control (500 ng/ml IGF-I in dilution buffer) was also included for subtraction. Standard, control, or samples (100 μl/well) were combined with 50 ng (100 μl) biotinylated goat anti-mouse IGF-II antibody (R&D) and incubated for 3 hr on a shaker at room temperature. The wells were washed three times with wash buffer, after which 200 μl of a 1:200 dilution of streptavidin-HRP conjugate (R&D) in dilution buffer was added to each well and incubated for 45 min at room temperature. The wells were then washed four times with wash buffer and 200 μl of 3,3′,5,5′-Tetramethylbenzidine in buffered hydrogen peroxide (Sigma) was added to each well and incubated at room temperature for 30 min to allow the color to develop. Then, 50 μl of 1 M H_2_SO_4_ was added per well to stop the reaction and produce a yellow color, and the absorbance was read at 450 nm.

## Results

### Generation and analysis of *Gga2^−/−^* compound heterozygotes

**Figure 1:**
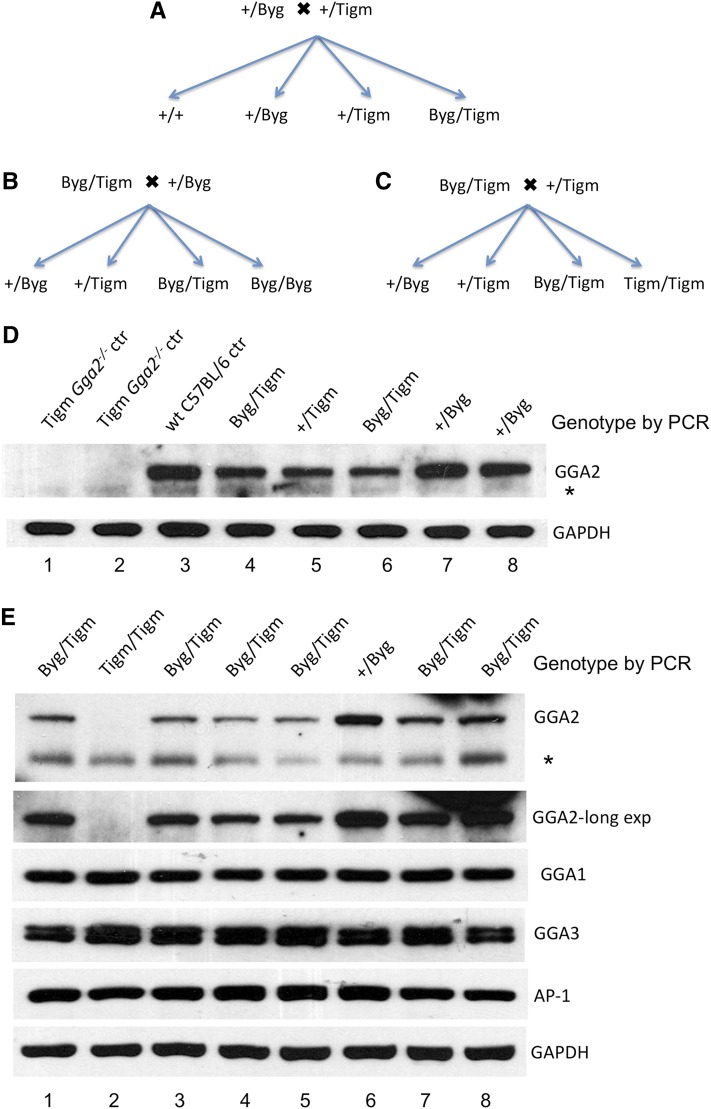
Generation and immunoblot analysis of Byg/Tigm compound hets. (A–C) Mating schemes used to generate compound heterozygous mice harboring both the Byg and Tigm mutant *Gga2* alleles. The Byg strain was backcrossed for at least eight generations into the C57BL/6 genetic background before being crossed with the Tigm strain, also in the C57BL/6 background. (D and E) Genotyping results and Western blot analysis of brain tissue from day 1 pups of representative litters resulting from the mating schemes shown in (B and C), respectively. Twenty-five μg of protein extract for each sample was subjected to SDS-PAGE and immunoblot analysis of GGA1, GGA2, GGA3, AP-1, and GAPDH (5 μg of lysate) as a control. *A non-specific band. Use of a 4–12% Bris-Tris gradient gel (E) resulted in better separation of the GGA2 band from the non-specific band compared to a 10% Tris-Glycine gel (D).

We initially crossed Byg *Gga2^+/−^* mice in the C57BL/6J genetic background with Tigm *Gga2^+/−^* mice in the C57BL/6NJ background using the mating scheme illustrated in Figure 1A. To facilitate the description of the compound heterozygotes, +/Byg and +/Tigm refer to mice that have one wild-type (wt) (+) and one *Gga2* gene-trap allele (either the Byg or the Tigm *Gga2* gene-trap allele, respectively), whereas Byg/Tigm are compound hets that harbor both the Byg and Tigm *Gga2* gene-trap alleles. Byg/Byg and Tigm/Tigm, however, refer to homozygous mice carrying two copies of either the Byg or the Tigm *Gga2* gene-trap alleles, respectively. Although the gene-trap cassette is inserted into intron 1 of the *Gga2* gene in both strains, the position of insertion was different (Figure S1), as was the nature of the vectors utilized. Details for genotyping that distinguished between the two alleles and allowed for identification of compound hets (Byg/Tigm) are provided in Figure S1. As expected if the Byg allele is hypomorphic, all the compound hets that were born were viable and normal in all respects even though these mice are *Gga2^−/−^* at the DNA level. We next mated the Byg/Tigm compound hets with either Byg *Gga2^+/−^* mice (+/Byg)( Figure 1B) or Tigm *Gga2^+/−^* (+/Tigm) (Figure 1C). The results of the genotyping and the Western blot data of the progeny (day 1 pups of a representative litter) from the former mating scheme are shown in Figure 1D. As controls, wt or Tigm *Gga2^−/−^* (Tigm/Tigm) samples were included. Strikingly, in this litter the two Byg *Gga2^+/−^* pups (Figure 1D +/Byg, lanes 7 and 8) had higher brain expression of GGA2 than the Tigm *Gga2^+/−^* pup (+/Tigm, lane 5) similar to wt (compare lane 3 with lanes 7 and 8). The two compound hets (Byg/Tigm, lanes 4 and 6), however, although genetically *Gga2^−/−^*, had similar expression as the Tigm *Gga2^+/−^* pup (+/Tigm, lane 5).

**Figure 2:**
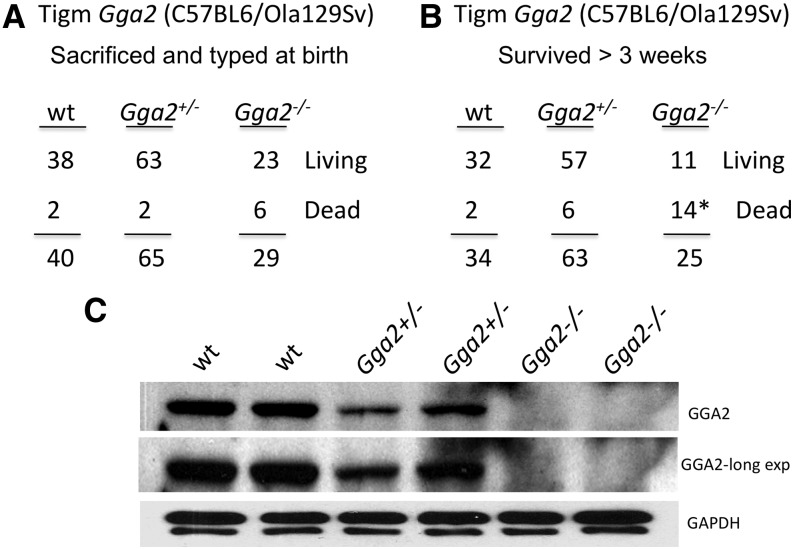
Influence of genetic background on severity of neonatal lethality associated with loss of GGA2. (A and B) The progeny of Tigm *Gga2^+/−^* crosses in the C57BL6/Ola129Sv mixed genetic background were genotyped by PCR within 24 hr after birth (A), or after 1 wk (B), and includes all dead pups that were recovered. The six deaths at birth of the *Gga2^−/−^* mice were not significant compared to the two wt deaths at birth, whereas the 14 deaths (*) that occurred neonatally were highly significant, as determined by the Fisher exact test (*P* < 0.0001). (C) Western blot analysis of brain tissue from representative day 1 pups resulting from Tigm *Gga2^+/−^* crosses in the mixed genetic background. Twenty-five μg of protein extract for each sample was subjected to SDS-PAGE and immunoblot analysis of GGA2 and GAPDH (5 μg of lysate) as a control.

We then examined brain extracts from another representative litter (Figure 1E) representing the mating scheme shown in Figure 1C. The large number of compound hets in this litter provided a good opportunity to monitor the GGA2 protein level in these mice. Immunoblot analysis of brain tissue extracts (Figure 1E) showed that the Tigm *Gga2^−/−^* pup in this litter (Tigm/Tigm, lane 2) had no detectable GGA2, even on long exposure of the blot, whereas the Byg *Gga2^+/−^* pup (+/Byg, lane 6) had the highest expression. Of note, GGA2 was detected in every compound het (Byg/Tigm, lanes 1, 3, 4, 5, 7, and 8), although there was substantial variability in the expression level (compare lanes 4 and 5 to 7 and 8.). It has been reported that a gene-trap inserted in intron 1 of the *Prep1* gene gave rise to a hypomorphic mutation that also resulted in variable expression of the Prep1 protein and an embryonic lethal penetrance that was dependent on the degree of leakiness of the gene-trap cassette (Ferretti *et al.* 2006). GGA1, GGA3, and AP-1, in contrast, did not display this variability between the different genotypes. Analysis of brain, heart, and lung tissue extracts from other litters also showed variation in the expression of GGA2 among the compound hets and Byg *Gga2^+/−^* (+/Byg) pups (Figure S2), indicating that the Byg allele must be producing wt message in addition to the chimeric *Gga2*-βgal transcript. The presence of this chimeric mRNA in Byg *Gga2^+/−^* mice was demonstrated previously by reverse-transcriptase polymerase chain reaction (RT-PCR) (Govero *et al.* 2012). Long exposure of the GGA2 blots also revealed that an occasional Tigm *Gga2^−/−^* pup expresses very low levels of GGA2 that could at times be detected (Figure S2, long exposure of brain and lung GGA2) but, for the most part, even if there was low level expression, it was below the detection limit (Figure 1E and Figure 2C long exposure of GGA2). Western blot analysis of varying amounts of wt brain lysate run alongside a *Gga2^−/−^* brain lysate where some expression was observed, indicating that this low level expression is in the range of 1–2.5% relative to wt (Figure S3). Importantly, progeny corresponding to every possible genotype were obtained from the crosses shown in Figure 1, A–C, with the exception of Byg *Gga2^−/−^* mice (Byg/Byg) (Figure 1B), highlighting the fact that the embryonic lethality associated with the Byg allele in the homozygous state persists in the C57BL/6NJ background.

These results confirm that the embryonic lethality of the Byg *Gga2^−/−^* mice is not attributable to the absence of GGA2. In view of this, we decided not to pursue this Byg strain further.

### Influence of genetic background on neonatal lethality of Tigm *Gga2^−/−^* gene-trap mice

We previously presented data showing that the Tigm *Gga2^−/−^* phenotype in the C57BL/6NJ background is neonatal lethal, exhibiting more than 95% mortality rate with one single survivor that lived beyond 3 wk (Govero *et al.* 2012). The surviving *Gga2^−/−^* mouse was a female that was 25% smaller than its littermates at 4 wk of age but had a normal lifespan. Genotyping of an additional 65 offspring from Tigm *Gga2^+/−^* intercrosses that survived beyond 2 wk identified only one *Gga2^−/−^* mouse that had survived up to that point. This surviving mouse was a male that weighed 50% less than its littermates and had to be killed at 3 wk due to marked cachexia. Histological examination did not reveal any pathologic changes in the internal organs (not shown), similar to the situation with the newborn *Gga2* knockouts (Govero *et al.* 2012). These results confirm our previous finding that *Gga2^−/−^* mice display a neonatal lethal phenotype in the C57BL/6NJ background.

To analyze the influence of the genetic background on the consequence of the loss of GGA2, the Tigm *Gga2* allele was introduced into the mixed background by mating Tigm *Gga2^+/−^* females in the C57BL/6NJ background with a wt male in the C57BL6/Ola129Sv mixed background. All the resulting N1 *Gga2^+/−^* mice were subsequently mated. Initially, all progeny of the *Gga2^+/−^* crosses were collected soon after birth and genotyped, including pups that died during or immediately after birth. Figure 2A shows that *Gga2^−/−^* mice in the mixed genetic background are born in near accordance with the expected Mendelian ratio, as was the case in the C57BL/6NJ background (Govero *et al.* 2012). Of note, the *Gga2^−/−^* mice exhibited increased mortality compared to wt and hets at this time point, although the difference was not statistically significant based on our sample size. In a separate experiment, 122 offspring were genotyped by PCR 1 wk after birth, along with the dead pups recovered during this period (Figure 2B). An additional three mice were recorded as being born but were missing and presumably cannibalized, so they could not be genotyped. Of the 25 *Gga2^−/−^* offspring that were born within this group, 14 (56%) died within the first week, with almost all dying within the first 48 hr. This is in stark contrast to the mortality rate of wt and *Gga2^+/−^* mice in the same background (6% and 10%, respectively). The result is also significantly different from the finding in the C57BL/6NJ background, where the lethality rate is more than 95% in the *Gga2^−/−^* homozygous state (Govero *et al.* 2012). Of the surviving Tigm *Gga2^−/−^* mice in the C57BL6/Ola129Sv mixed background, none have died beyond the 3-wk period, with the oldest mouse now 9 months old, and both males and females are fertile. Because 11 of 25 Tigm *Gga2^−/−^* mice in the mixed background survived beyond 3 wk, it was conceivable that these mice might express more GGA2 than do the Tigm *Gga2^−/−^* mice in the C57BL/6NJ background. However, Western blot analysis of brain tissue extracts prepared from day 1 pups in the mixed background probed for GGA2 showed no detectable protein in the *Gga2^−/−^* mice in most instances (representative pups shown in Figure 2C), although occasionally the same low-level expression was observed as with the Tigm *Gga2^−/−^* mice in the C57BL/6NJ background. Taken together, these results indicate that the partial rescue of the neonatal lethality in the mixed background is not due to increased expression of GGA2, but rather it is the genetic background of the mice that plays an important role in determining viability in the absence or near-complete loss of GGA2.

**Figure 3:**
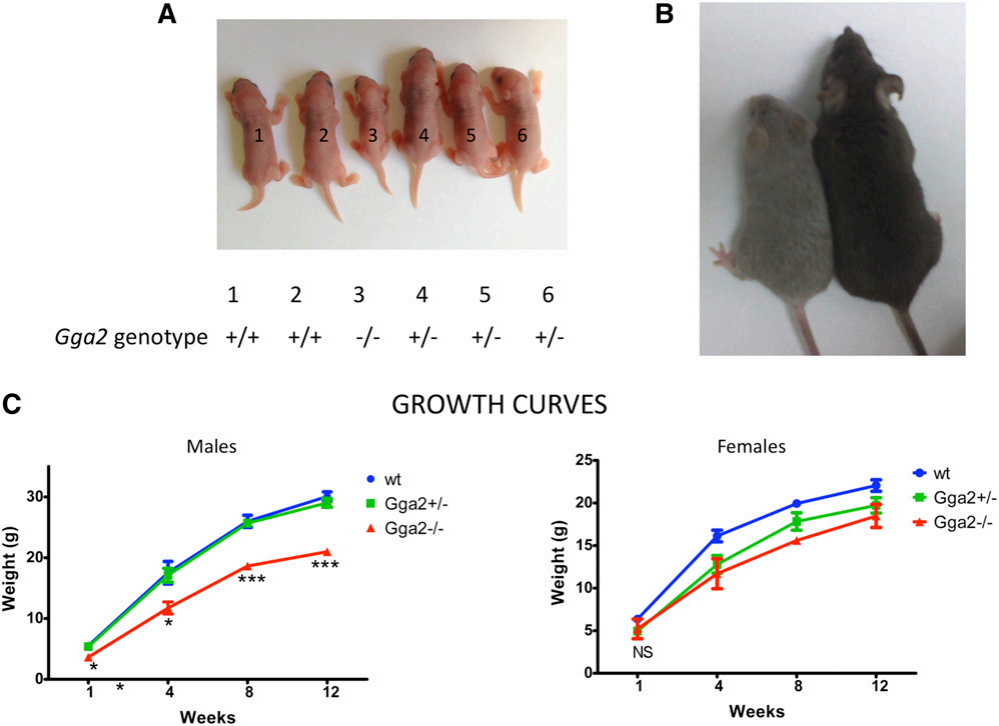
Growth retardation of *Gga2^−/−^* mice. (A) Picture of day 1 pups from a single litter of a Tigm *Gga2^+/−^* cross in the mixed genetic background shows that the homozygous knockout has a markedly reduced body size. (B) The male *Gga2^−/−^* mouse (left mouse) in the mixed genetic background is much smaller than its female wt littermate at 6 wk of age but is otherwise healthy. (C) Weights were obtained from week 1 to week 12 and plotted according to gender and genotype. Results shown are mean ± SEM for each time point. Males: wt, n = 7; *Gga2^+/−^*, n = 11; *Gga2^−/−^*, n = 6. Females: wt, n = 3; *Gga2^+/−^*, n = 2; *Gga2^−/−^*, n = 2. *P* values for pairwise comparisons of mean weights between wt and *Gga2^−/−^* male mice are as follows: *P < 0.05, **P < 0.005, and ***P < 0.0005. NS, not significant. Statistical analysis was not performed with the females due to the small sample size.

**Figure 4:**
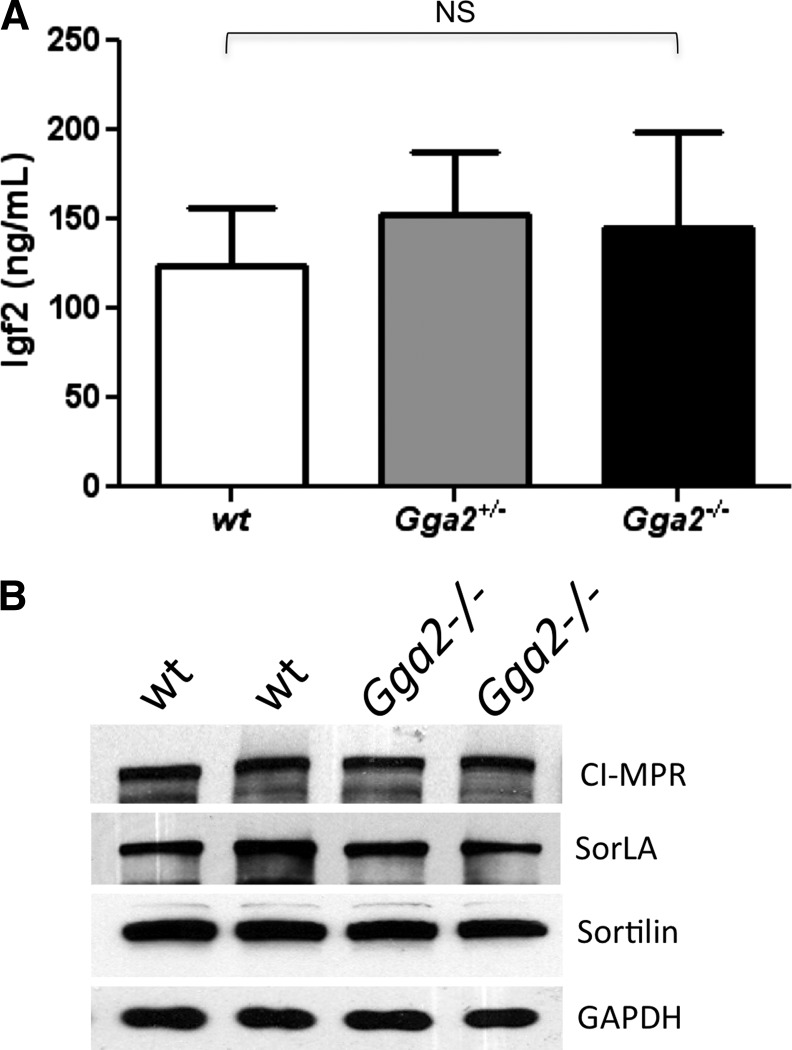
Analysis of serum IGF-II and brain IGF-II receptor (CI-MPR) levels. (A) Serum IGF-II levels of newborn mice were determined by ELISA as described in *Materials and Methods*. The serum obtained from each pup was sufficient to perform the assay in duplicate. Results are mean ± SD, n = 16 per group. (B) Western blot analysis of brain tissue from two wt and two Tigm *Gga2^−/−^* mice in the mixed background. Twenty-five μg protein extract for each sample was subjected to SDS-PAGE and immunoblot analysis of CI-MPR, SorLA, and Sortilin. GAPDH (5 μg of lysate) was included as a loading control. NS, not significant as determined by Student *t* test.

One consistent phenotype that was observed with the *Gga2^−/−^* mice in the mixed genetic background was that both males and females were significantly smaller than their siblings (Figure 3, A–C). Because the GGAs are known to be involved in the trafficking of the cation-independent mannose 6-phosphate receptor (CI-MPR) (Nielsen *et al.* 2001; Puertollano *et al.* 2001; Takatsu *et al.* 2001; Zhu *et al.* 2001), which clears insulin-like growth factor-II (IGF-II) from blood (Morgan *et al.* 1987), we considered the possibility that loss of GGA2 might result in increased levels of the CI-MPR at the plasma membrane, which, in turn, might lower the blood IGF-II level and inhibit growth. To investigate this possibility, serum IGF-II levels of day 1 pups were measured by enzyme- linked immunosorbent assay (ELISA). The levels were similar in all three genotypes, indicating that lack of circulating IGF-II is not the cause of the reduced body weight of *Gga2^−/−^* mice (Figure 4A). We also found that the expression level of the CI-MPR, SorLA, and Sortilin are unaltered in brain tissue of *Gga2^−/−^* mice (Figure 4B), excluding major differences in the turnover of these receptors in the absence of GGA2. Moreover, plasma levels of four different lysosomal acid hydrolases were assayed and found to be similar between wt and *Gga2^−/−^* mice (not shown), indicating that acid hydrolase sorting is normal in the absence of GGA2.

## Discussion

The data presented here extend and clarify our previous report concerning the consequences of *Gga2* gene-trap disruption (Govero *et al.* 2012). We reported that the Byg *Gga2^−/−^* mice in both the mixed and C57BL/6J genetic backgrounds exhibit early embryonic lethality, whereas the Tigm *Gga2^−/−^* mice in the C57BL/6NJ background display neonatal lethality. The current experiments unequivocally demonstrate that the early embryonic lethality of the Byg *Gga2^−/−^* mice is not due the absence of GGA2 because the Byg *Gga2* gene-trap allele is clearly hypomorphic. Because the *Gga1* and *Gga3* genes were also disrupted using the same vector as in the Byg *Gga2* strain and the corresponding gene-trap homozygotes are complete knockouts (Govero *et al.* 2012), this result was unexpected. However, similar phenomena have been reported for a number of other trapped genes, including the iron regulatory protein *(IRP)-1* and *IRP-2* genes where the pre-mRNAs transcribed from identical gene-trap cassettes are processed very differently within the two different gene contexts, with the IRP1 mRNA being undetectable while the IRP2 mRNA was unchanged (Galy *et al.* 2004). In the case of the Byg *Gga2* gene-trap allele, this alternate splicing around the gene-trap cassette in many instances gives rise to near-normal levels of GGA2, showing that the early embryonic lethality is the consequence of another factor that has yet to be identified. Based on the finding with the Tigm *Gga2^−/−^* mice, we conclude that GGA2 is important for viability of neonates but appears to be dispensable once the mice are past 3 wk of age. In this regard, the genetic background has a major impact in terms of the tolerance to the loss of GGA2, with more than 95% lethality occurring in the C57BL/6NJ background (Govero *et al.* 2012) compared with 56% in the C57BL6/Ola129Sv mixed genetic background. This difference is not due to increased expression of GGA2 in the mixed background. A similar phenotype has been reported for *Nedd4-2^−/−^* mice, in which the targeted homozygous nulls in the C57BL/6J background exhibit almost complete neonatal lethality, which was partially rescued by crossing into the same mixed genetic background as our mice (Boase *et al.* 2011). In addition, a number of other gene knockouts in mice have been shown to present considerable variation in the penetrance of lethality, depending on the genetic background in which the knockouts were made (Doetschman 2009).

In our previous study, it was shown that single knockouts of *Gga1* and *Gga3* in the mixed genetic background are, for the most part, phenotypically normal (Govero *et al.* 2012). In addition, we reported that *Gga1^−/−^* mice in the C57BL/6J background are viable. We have now backcrossed *Gga3^−/−^* mice into the C57BL/6J background as well and have found no obvious difference between the two genetic backgrounds with either knockout strain, indicating that loss of GGA1 or GGA3 is well-tolerated in both strains, in contrast to the loss of GGA2. We have shown that even though brain expression of the three GGAs was detected at all stages of development, GGA2 expression was highest at embryonic day 9 (day E9) through the end of week 1, and then declined substantially reaching the lowest level in the adult mouse brain (Govero *et al.* 2012). Brain expression levels of GGA1 and GGA3, however, remained consistently high from day E9 through the adult stage (Govero *et al.* 2012). These findings are in concordance with some non-redundant and vital function(s) mediated by GGA2 early in the life of a mouse. This is not to imply that GGA1 and GGA3 together are completely dispensable for survival. Our previous study demonstrated that GGA1/GGA3 double knockout mice show 58% neonatal lethality in the mixed genetic background, similar to the 56% lethality due to loss of GGA2 in the same background. What are the overlapping compared to distinct physiological roles served by GGA1/GGA3 compared to GGA2? This is an important question that warrants future investigation.

## 

## Supplementary Material

Supporting Information
