# Supplementary material for: Impact of Genetic Background on Neonatal Lethality of Gga2 Gene-Trap Mice
Source: G3 (Bethesda). 2014 Mar 17;4(5):885–90. doi: 10.1534/g3.114.010355 (PMC4025487; doi:10.1534/g3.114.010355)
Supplement: Supporting Information [file supp_4_5_885__index.html]

Impact of Genetic Background on Neonatal Lethality of Gga2 Gene-Trap Mice — Supporting Information 

# Impact of Genetic Background on Neonatal Lethality of *Gga2* Gene-Trap Mice

## Supporting Information for Doray, Govero, and Kornfeld, 2014

**Files in this Data Supplement:**

- Supporting Information - Figures S1-S3 (PDF, 287 KB)
- Figure S1 - Genotyping strategy for identifying compound heterozygotes. (PDF, 146 KB)
- Figure S2 - Tissue expression of GGA2 in mice carrying the Byg allele. (PDF, 166 KB)
- Figure S3 - Detection limit of GGA2 in brain lysates obtained from mixed background mice. (PDF, 147 KB)
