# Supplementary material for: Impact of Genetic Background on Neonatal Lethality of Gga2 Gene-Trap Mice
Source: G3 (Bethesda). 2014 Mar 17;4(5):885–90. doi: 10.1534/g3.114.010355 (PMC4025487; doi:10.1534/g3.114.010355)
Supplement: Supporting Information [file supp_g3.114.010355_010355SI.pdf]

G3 · Genes | Genomes | Genetics

Impact of genetic background on neonatal lethality of *Gga2* knockout mice

Balraj Doray[1], Jennifer Govero[1] and Stuart Kornfeld[1,*]

[1]Department of Internal Medicine, Washington University School of Medicine, St. Louis, MO 63110, USA.

*To whom correspondence should be addressed. Mailing address: 660 S. Euclid Ave, Campus Box 8125, Department of Internal Medicine, Washington University School of Medicine, St. Louis, MO 63110. E-mail: skornfel@dom.wustl.edu. Phone: (314)- 362-8803. Fax: (314)-362-8826.

DOI: 10.1534/g3.114.010355

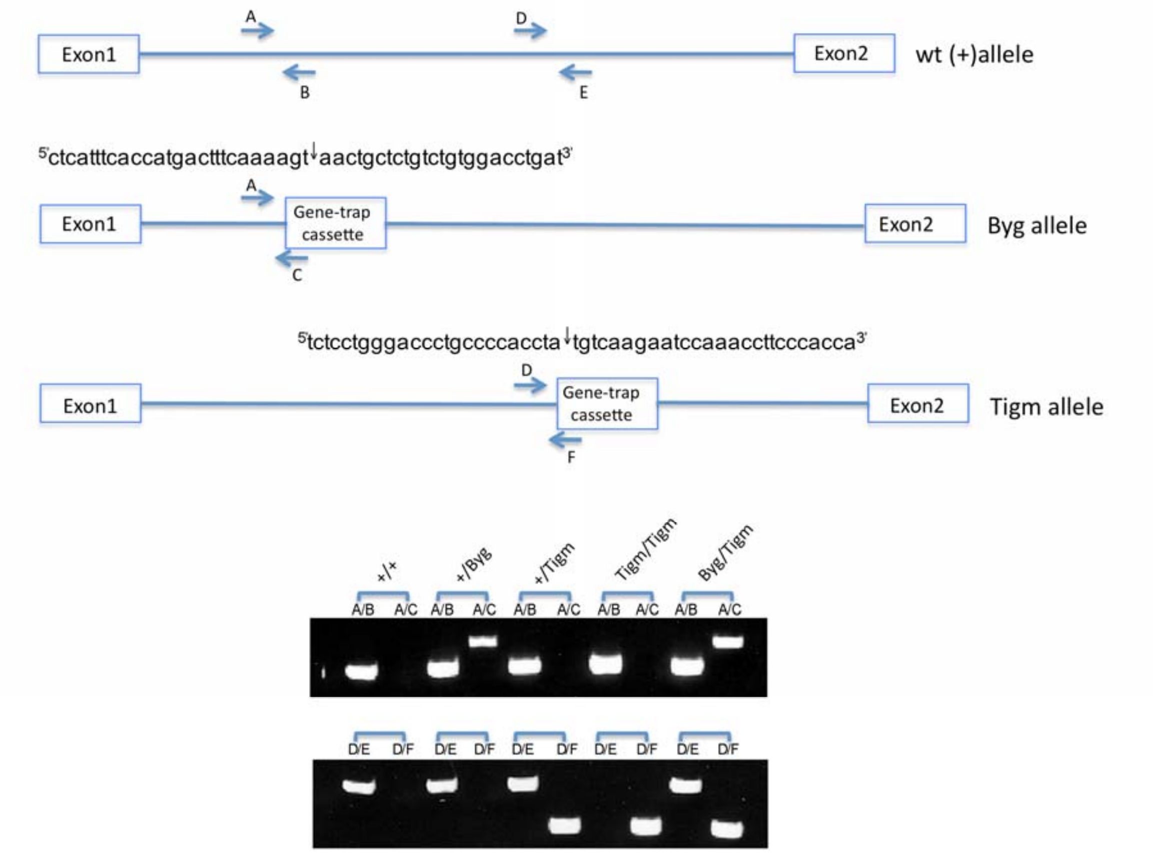

Figure S1 Genotyping strategy for identifying compound heterozygotes. Compound heterozygotes harbor 2 different gene-trapped *Gga2* alleles, distinguished from each other by use of the various primer pairs (A/B, A/C, D/E and D/F) shown here. Fifty nucleotides of intronic sequence around each gene-trap cassette are shown. Arrow indicates site of insertion. In the case of the wt allele, only primer sets A/B and D/E will yield PCR products of the correct size. In the presence of the Byg allele, only primer sets A/C and D/E, but not A/B and D/F, will give the correct PCR products. In the presence of the Tigm allele, only primer sets A/B and D/F, but not A/C and D/E, will give the correct PCR products. In the case of the compound het where one copy each of the Byg and Tigm alleles are present, all four primer sets, A/B, A/C, D/E and D/F will yield correct PCR products. Results for Byg/Byg are not shown since no pups having two Byg alleles (Byg/Byg) were ever born.

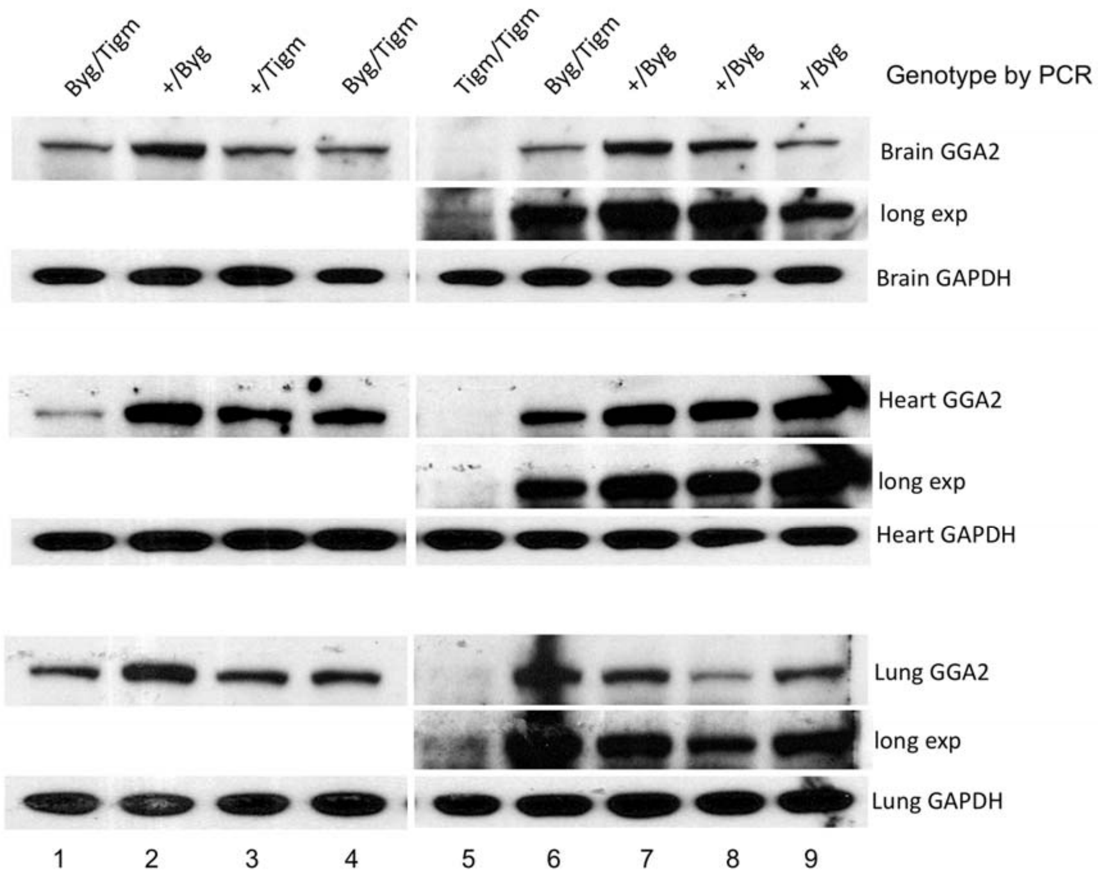

Figure S2 Tissue expression of GGA2 in mice carrying the Byg allele. Samples in lanes 1-4 and 5-9 are from pups resulting from the mating schemes shown in Figures 1B and 1C, respectively. 25 μg of protein extract for each sample from the different tissues was subjected to SDS-PAGE and immunoblot analysis of GGA2 and GAPDH (5 μg of lysate) as a control.

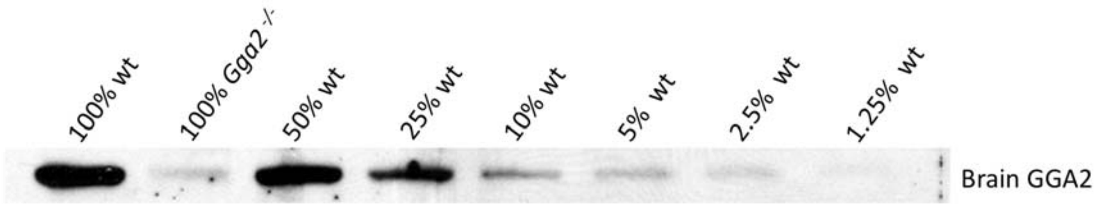

Figure S3 Detection limit of GGA2 in brain lysates obtained from mixed background mice. 40 µg of wt or *Gga2*$^{-/-}$ lysate (100%) were loaded alongside 50%, 25%. 10%, 5%, 2.5% and 1.25% of wt lysate, and subjected to SDS-PAGE and immunoblot analysis of GGA2.
