# Supplementary material for: Impact of Genetic Background on Neonatal Lethality of Gga2 Gene-Trap Mice
Source: G3 (Bethesda). 2014 Mar 17;4(5):885–90. doi: 10.1534/g3.114.010355 (PMC4025487; doi:10.1534/g3.114.010355)
Supplement: Supporting Information [file supp_g3.114.010355_FigureS1.pdf]

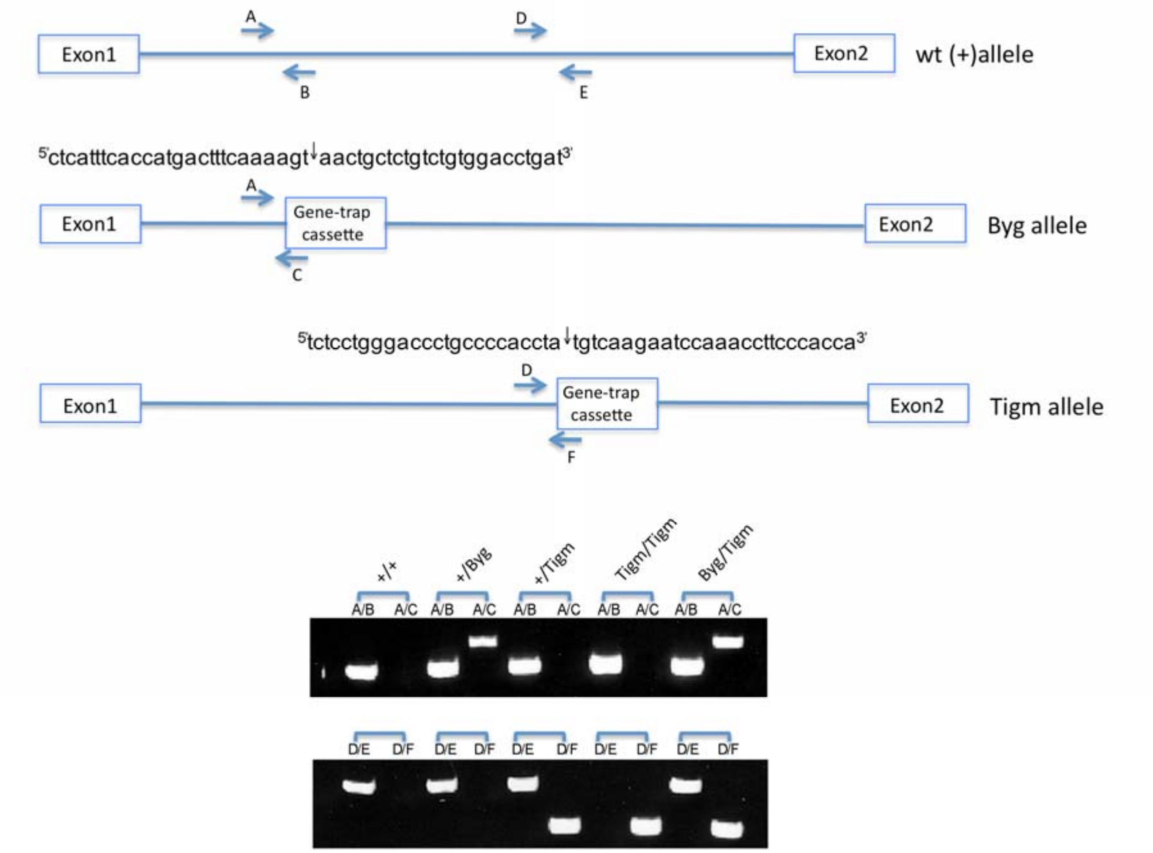

Figure S1 Genotyping strategy for identifying compound heterozygotes. Compound heterozygotes harbor 2 different gene-trapped *Gga2* alleles, distinguished from each other by use of the various primer pairs (A/B, A/C, D/E and D/F) shown here. Fifty nucleotides of intronic sequence around each gene-trap cassette are shown. Arrow indicates site of insertion. In the case of the wt allele, only primer sets A/B and D/E will yield PCR products of the correct size. In the presence of the Byg allele, only primer sets A/C and D/E, but not A/B and D/F, will give the correct PCR products. In the presence of the Tigm allele, only primer sets A/B and D/F, but not A/C and D/E, will give the correct PCR products. In the case of the compound het where one copy each of the Byg and Tigm alleles are present, all four primer sets, A/B, A/C, D/E and D/F will yield correct PCR products. Results for Byg/Byg are not shown since no pups having two Byg alleles (Byg/Byg) were ever born.
