# Supplementary material for: Impact of Genetic Background on Neonatal Lethality of Gga2 Gene-Trap Mice
Source: G3 (Bethesda). 2014 Mar 17;4(5):885–90. doi: 10.1534/g3.114.010355 (PMC4025487; doi:10.1534/g3.114.010355)
Supplement: Supporting Information [file supp_g3.114.010355_FigureS2.pdf]

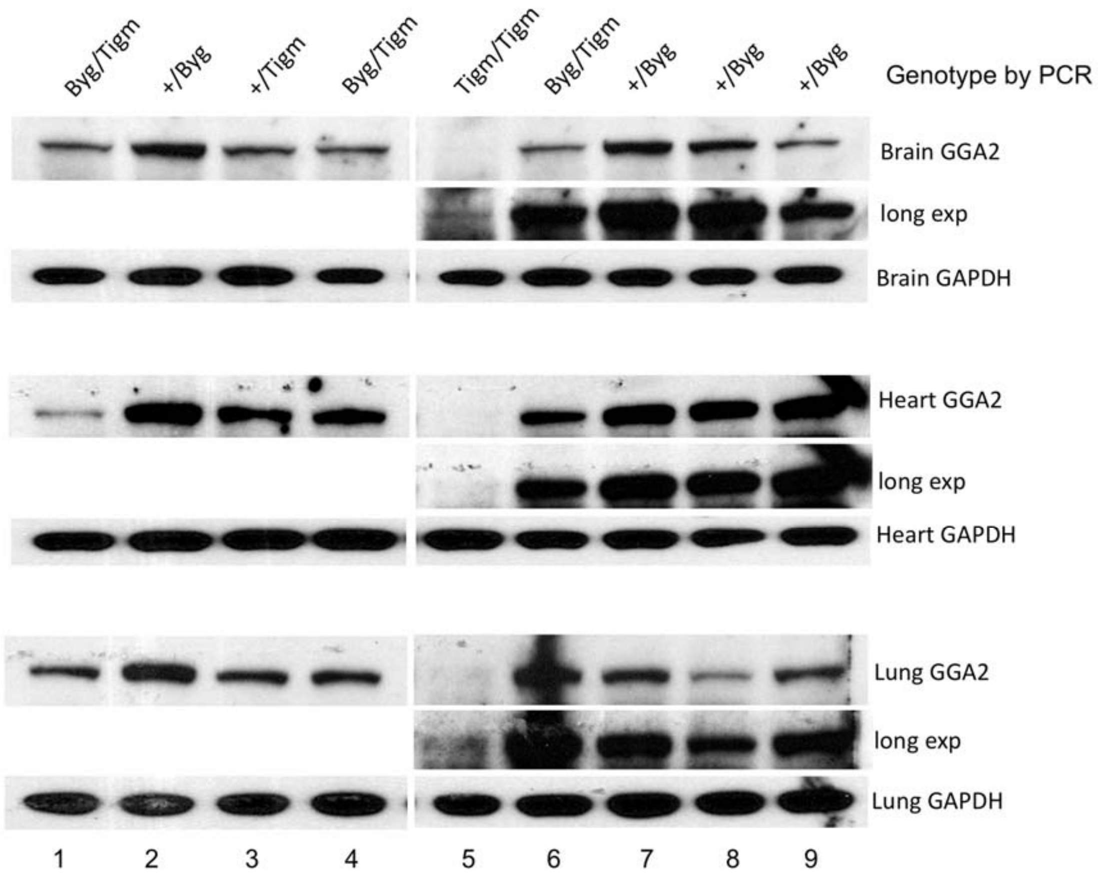

Figure S2 Tissue expression of GGA2 in mice carrying the Byg allele. Samples in lanes 1-4 and 5-9 are from pups resulting from the mating schemes shown in Figures 1B and 1C, respectively. 25 μg of protein extract for each sample from the different tissues was subjected to SDS-PAGE and immunoblot analysis of GGA2 and GAPDH (5 μg of lysate) as a control.
