# Supplementary material for: Impact of Genetic Background on Neonatal Lethality of Gga2 Gene-Trap Mice
Source: G3 (Bethesda). 2014 Mar 17;4(5):885–90. doi: 10.1534/g3.114.010355 (PMC4025487; doi:10.1534/g3.114.010355)
Supplement: Supporting Information [file supp_g3.114.010355_FigureS3.pdf]

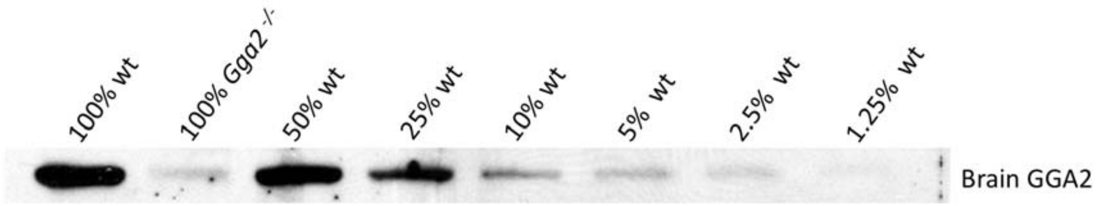


**Figure S3 Detection limit of GGA2 in brain lysates obtained from mixed background mice.** 40 μg of wt or *Gga2*$^{-/-}$ lysate (100%) were loaded alongside 50%, 25%. 10%, 5%, 2.5% and 1.25% of wt lysate, and subjected to SDS-PAGE and immunoblot analysis of GGA2.
